# Protistan epibionts affect prey selectivity patterns and vulnerability to predation in a cyclopoid copepod

**DOI:** 10.1038/s41598-022-26004-5

**Published:** 2022-12-31

**Authors:** Ram Kumar, Suman Kumari, Anshu Malika, A. P. Sharma, Hans-Uwe Dahms

**Affiliations:** 1 Department of Environmental Science, Central University of South Bihar, Gaya, Bihar 824326 India; 2 ICAR-Central Inland Fisheries Research Institute, Barrackpore, Kolkata, 700120 India; 3 Govind Ballabh Pant University of Agriculture and Technology, Udham Singh Nagar, Pantnagar, Uttarakhand 263155 India; 4 Department of Biomedical Science and Environmental Biology, College of Life Science, Kaohsiung Medical University, Kaohsiung, 80424 Taiwan

**Keywords:** Ecology, Environmental sciences, Limnology

## Abstract

Colonisation of crustacean zooplankton with ciliate epibionts is widespread in freshwater and marine environments. However, the ecology of such association are little studied as yet. The occurrence of ciliate epibionts on copepods and the preference towards this association with different life stages of *Mesocyclops* were studied from winter to spring. Relative susceptibility of zooplankton species was evaluated by analysing the epibiont colonies and zooids and relate this to the surface area of the host. The maximum epibiont infestation per unit body surface area was recorded on copepodites followed by copepod nauplii rather than other zooplankton species, whereas the rotifer *Asplanchna* was never affected. Influence of climatic factors such as temperature on the colonisation of epibionts on basibionts was found significant. In winter (November to February) samples, copepods were infested by autotrophic epibionts whereas in late spring and early summer (March–April) heterotrophic protists (peritrichian ciliates) were the sole epibionts on copepods. We conducted experiments in the laboratory on prey selection pattern of predators by direct visual and video-graphic observations of various events (encounter, attack, capture, ingestion, prey escape) during predation by infested and uninfested copepodites and adults of *Mesocyclops*. Postencounter the attack probability was significantly lower in infested than in uninfested copepods. The present paper reports on substrate preference by epibionts and their impacts in food rich and food scarce environments. Furthermore, major environmental interactions were studied with the reproductive phenology of copepods with respect to epibionts and the cause and effect of long term association of epibionts with copepods need to be addressed.

## Introduction

Epibiosis, the colonisation of a living surface, represents the association of two organisms: the epibiont, accommodated by a basibiont host^1,2^. Several commensal interactions were reported from epibiotic associations but there are also reports showing that epibionts can cause negative effects like ontogenetic or behavioural changes of the basibiont^3^. Fernandez-Leborans analysed different aspects of ecological, physiological and evolutionary associations^4^. A sessile organism is benefitted while attached to a living, motile host surface in terms of expense-free gene flow, transportation to favourable zones and protection from predation^5,6^. However, the colonisation driven changes on the body surface of the basibionts may cause different effects including prey capture success, escape from predation, sinking rates, increasing susceptibility to visual predators, decreasing darting efficiency and swimming speed, decreasing mating success and survival probability^7^. On the other hand, basibionts can be benefitted by the external covering of body surface, insulating coatings of the cuticles. The outer body surface of an organism represents its major physiological interface with the ambient environment, which play a direct role in entry and exit of substances during respiration, exudation of wastes and secondary metabolites, irradiation and various infochemicals. From an ecological perspective, most responses of an organism to environmental stressors, kairomones, infochemicals, irradiation, predators, parasites and signals from conspecifics are mainly the function of the external body surface. So colonisation by epibionts is likely to harm zooplankton by blocking receptors and protect zooplankton from environmental stressors, invertebrate predators (contributed by larger body size) and radiation.

These could be opportunistic, facultative, and non-specialized, with epibionts also colonizing inert substrates, animals and plants. But it could be also an obligatory and highly specific association, with morphological and behavioural adaptations between epibiont, basibiont, and the ambient environment^5^.

Within the plankton community, ciliated protists, certain mixotrophic flagellates and rotifers (*Brachionus rubens*) are frequently observed epibionts on other zooplankton^8–10^. Most of the studies on epibiosis in planktonic communities are confined to systematics or structural content focussing on their spatio-temporal distribution. Very few have considered other ecological aspects^11–14^. A number of ciliated protists colonize as epibionts various zooplankton species^4,9,14,15^. Recent studies tend to focus on adverse effects of epibionts on the host, such as decreased fecundity, interference with feeding, and locomotion, and increased sensitivity to contaminants^10,15–19^. Among ciliated protists, apostomatids, peritrichs, suctorians, heterotrichs, and chonotrichs are reported as epibionts on rotifers and crustaceans^4,19^. There are some anecdotal studies that describe the role of ciliate epibionts on hydrozoans, bryozoans, but the role of ciliates as epibionts of zooplankton has not been elucidated convincingly. A total of 30 genera of peritrichs have been described as epibionts among 5 major groups of crustaceans^19^. The ecological interactions of epibionts with respect to prey predator relationships especially from tropical and sub-tropical ecosystems is little known^1,4,11,17,20^.

Zooplankton communities possess diverse feeding modes, swimming patterns, multiple foraging strategies and predation escape behaviour. They are simultaneously subjected to both, tactile invertebrate and visual fish predation pressures. So, epibionts on zooplankton are likely to affect different zooplankton species differentially based on foraging strategy, swimming patterns, body surface morphology and predation pressure^21^.

The association of protistan phototrophic epibionts, with the freshwater cyclopoid copepod *Mesocyclops* sp. are wide-spread in freshwater ecosystem, but available documents on their role in ecology, such as susceptibility of *Mesocyclops* sp. and their predation is scanty. In the present paper we attempt to solve the intriguing problem how epibionts affect basibionts in terms of feeding, mobility and being preyed upon”.

We tested the following null hypotheses: (i) Since they have a carapace and joint appendages copepods would be more susceptible to epibionts than rotifers in a natural setting; (ii) predatory copepods loaded with epibionts would require higher energy and prefer larger prey than conspecific individuals without epibionts; (iii) visual predators like planktivorous fish would selectively utilize the prey loaded with epibionts as predicted by optimal foraging models.

## Results

**Figure 1:**
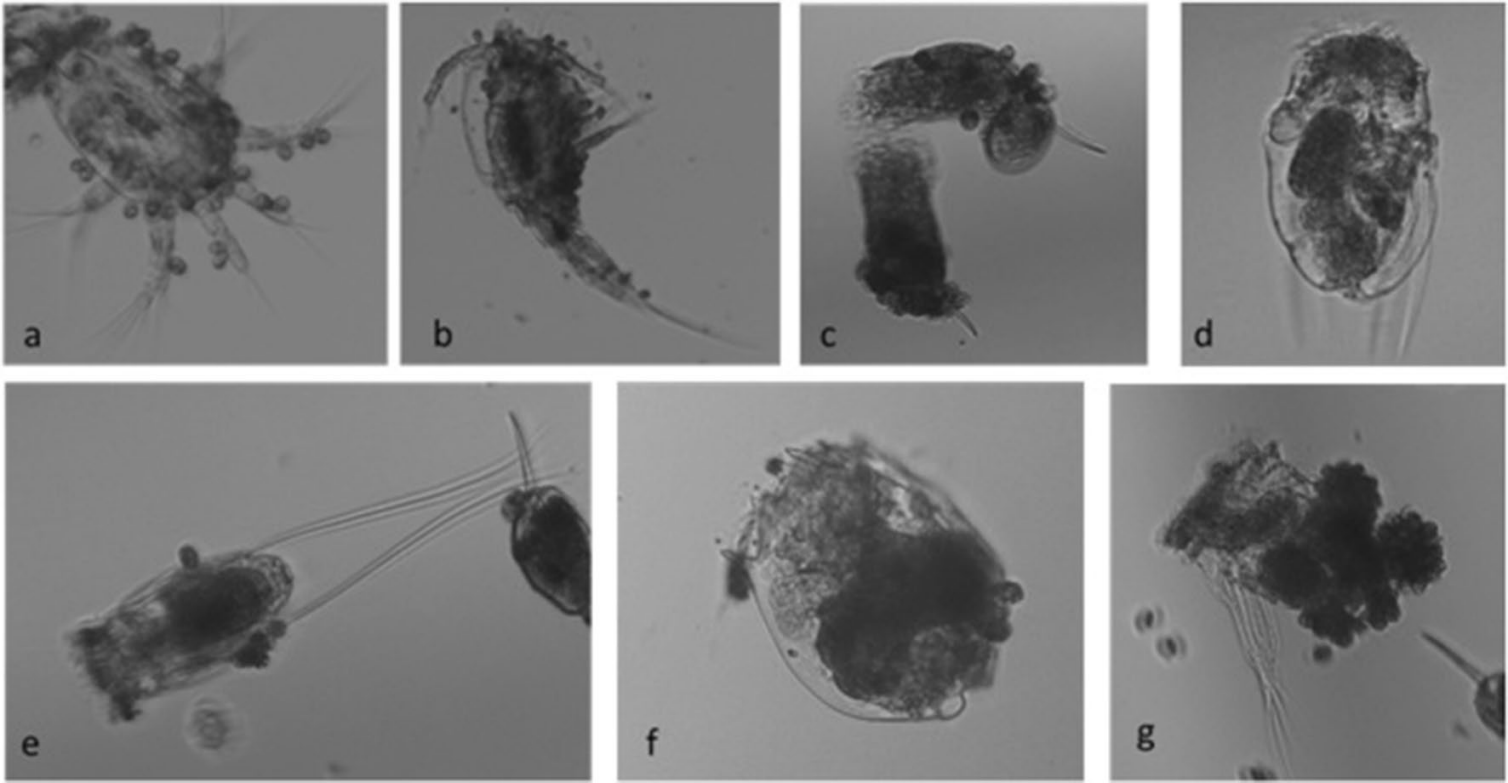
Zooplankton species loaded with epibionts (**a**) Copepod nauplii (**b**) Copepod adults (**c**) *Keratella cochlearis* (**d**) *Polyarthra vulgaris* (**e**) *Filinia longiseta* (**f**) *Brachionus rubens* (**g**) *Hexarthra mira*.

**Figure 2:**
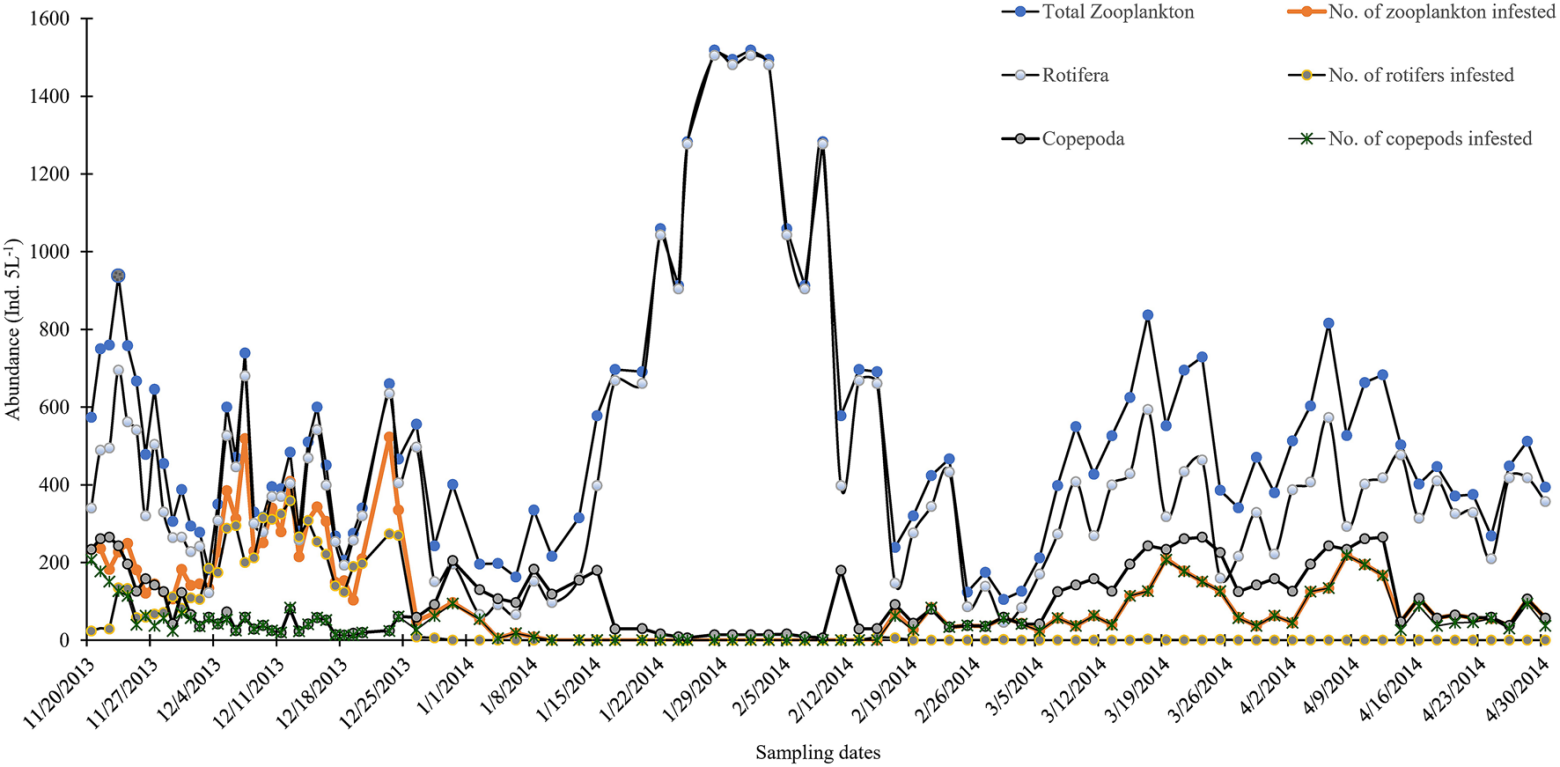
Total abundance and individual numbers colonized (individuals 5 L^−1^) by epibionts at each sampling date during November 2013 to April 2014, from the flood plain wetland of the river Ganges.

Environmental factors such as temperature, alkalinity, hardness, DO, pH and chlorophyll content were provided in supplementary table (Supplementary Table S1). Ciliate epibiont associations were observed on all rotifers, copepod adults, copepodids and nauplii (Fig. 1). Assessment of epibiont infestation on zooplankton was carried out from 20th November 2013 to 30th April 2014 (Fig. 2). Except during the 1st week of January to the 1st week of February different zooplankton species were observed loaded with differential numbers of epibionts (≤ 53 epibionts Ind^−1^). During this period 20–70% of zooplankton were carrying epibionts (Fig. 2). Both colonial and solitary forms of epibionts were recorded on the body surface of copepods- adults and copepodids, on the body surface of *Keratella* sp, whereas on copepod nauplii and other rotifers only solitary forms were recorded. We recorded a total of three genera of epibiotic protista including ciliates (*Rhabdostyla* Kent, 1880 and *Epistylis* Ehrenberg, 1830) and the mixotrophic flagellates (*Colacium vesiculosum* Ehrenberg, 1853).

**Figure 3:**
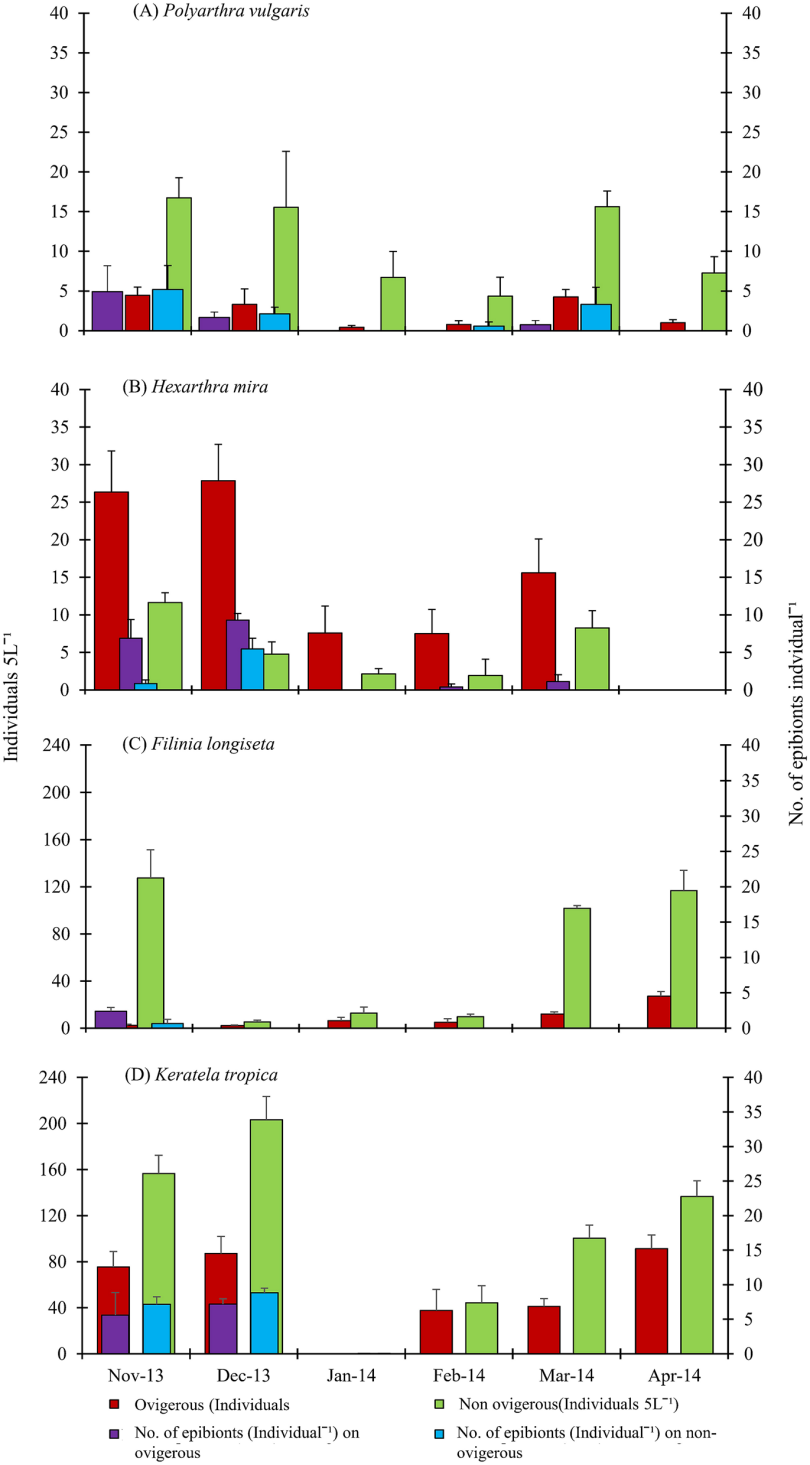
Monthly average of abundance (Individuals 5L^−1^ and No. of epibionts Individual^−1^ of ovigerous and non-ovigerous rotifers (**A**) *Polyarthra*, (**B**) *Hexarthra* (**C**) *Filinia* and (**D**) *Keratella* during November 2013 to April 2014, from the flood plain wetland of the river Ganges.

**Figure 4:**
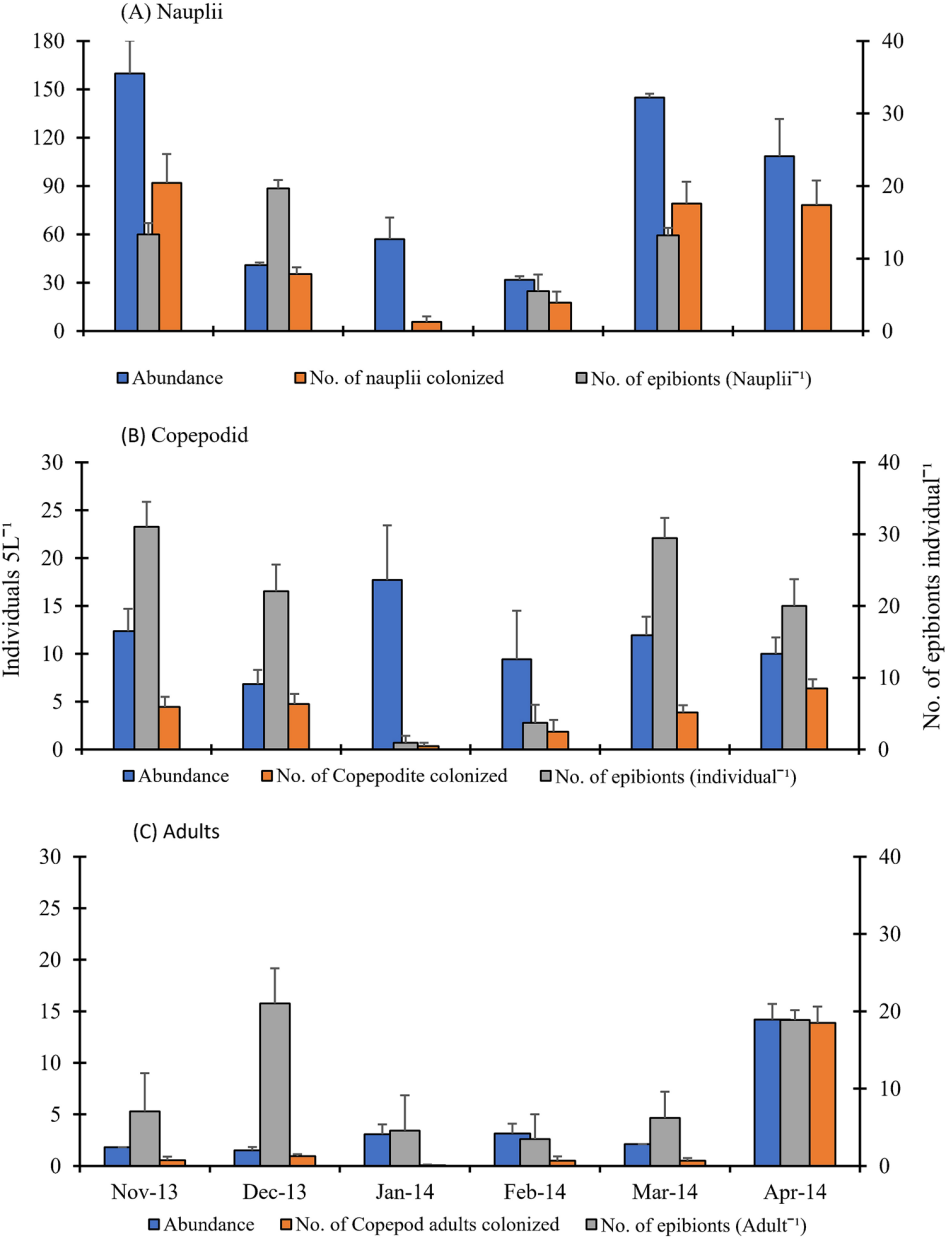
Monthly average of abundance (Individuals 5L^−1^) and infestation load (No. of epibiont individual^−1^) of ovigerous and non-ovigerous copepods, *Mesocyclops* (**A**) nauplii, (**B**) copepodid and (**C**) adults during November 2013 to April 2014, from the flood plain wetland of the river Ganges.

The number of epibionts varied from species to species of the group of zooplankton as shown in (Figs. 3 and 4). Association of epibionts were observed highest in copepods (*Mesocyclops* sp.) (Fig. 4) followed by nauplii and rotifers. Interestingly, *Asplanchna* was found free of epibiont infestation throughout the study period.

Colonisation by epibionts was observed on *Ceriodaphnia, Brachionus* and *Polyartha.* But no ovigerous female *Ceriodaphnia* was observed throughout the study period. Infestation load was recorded on all stages of copepods including males, ovigerous and non-ovigerous females, copepodids, and nauplii. The species–specific frequency distribution of epibionts for zooplankton species was ≥ 5% population where one or more showed epibionts. In the present study zooplankton species loaded with epibionts were observed in some rotifer species such as *Keratella, Hexarthra*, *Polyarthra*, *Filinia,* and *Brachionus* adults ovigerous and non-ovigerous were also found with differential loads of epibionts (Figs. 1, 3). Copepod adults ovigerous and nonovigerous both, their developmental stages -nauplii and copepodid stages (Fig. 4) were heavily infested (up to 53 epibionts Ind^−1^). Infestation levels (no. of epibionts basibiont^−1^) on copepod nauplii, copepod adults, *Keratella cochlearis*, *Polyarthra vulgaris, Filinia longiseta*, *Brachionus rubens* and *Hexarthra mira* are given in Figs. 3 and 4. In consideration with per unit surface area the infestation load on evasive rotifer *P. vulgaris* was maximal, followed by *H. mira* and *K. cochlearis* whereas among copepods juvenile stages were more susceptible than adult copepods. Epibiont density per unit surface area of the basibionts ranged from 0.73 in *B. rubens* to 207.3 cells mm^−2^ in *P. vulgaris.*

Relative susceptibility of per individual zooplankton to epibiont infestation was highest in case of males, copepodids, nauplii followed by non-ovigerous female adult cyclopoids. Ovigerous copepods were found less susceptible to epibionts (Fig. 4) than other copepod life stages. However, in terms of per unit body surface area copepod nauplii and copepodids were more loaded with epibionts than adult copepods.

The prevalence of epibiont numbers on ovigerous females, copepodids and nauplii was correlated with temperature (range: 16.5–31.5 °C; mean ± SE: 22.6 ± 2.25). In general, 2 to 53 epibionts were recorded on each individual zooplankter during this study. A maximum of 53 individuals of epibionts were recorded on a copepodid body on 21st November 2013. Average numbers of epibionts recorded per individual rotifer as provided in Fig. 3, that for each individual copepod of different developmental stages is provided in Fig. 4. Significantly lower numbers of epibionts were observed on all developmental copepod stages in February 2014 (Mann Whitney *U* test). The swimming movements in infested and uninfested adults and copepodids of *Mesocyslops* were monitored in 90 mm diameter petridishes with a bottom-grid composed of 1 × 1 cm cells. Swimming paths were traced and numbers of power strokes (leaping) were recorded and repeated for one minute, observations for at least five individuals of infested and uninfested adults and copepdid stages of *Mesocyslops*. No. of power strokes for copepodids were significantly higher than those of adult *Mesocyslops* and displacement rates were significantly lower in infested copepodids and adults than the corresponding uninfested individuals.

### Effects of epibiosis on predation by basibiont (copepod)

**Figure 5:**
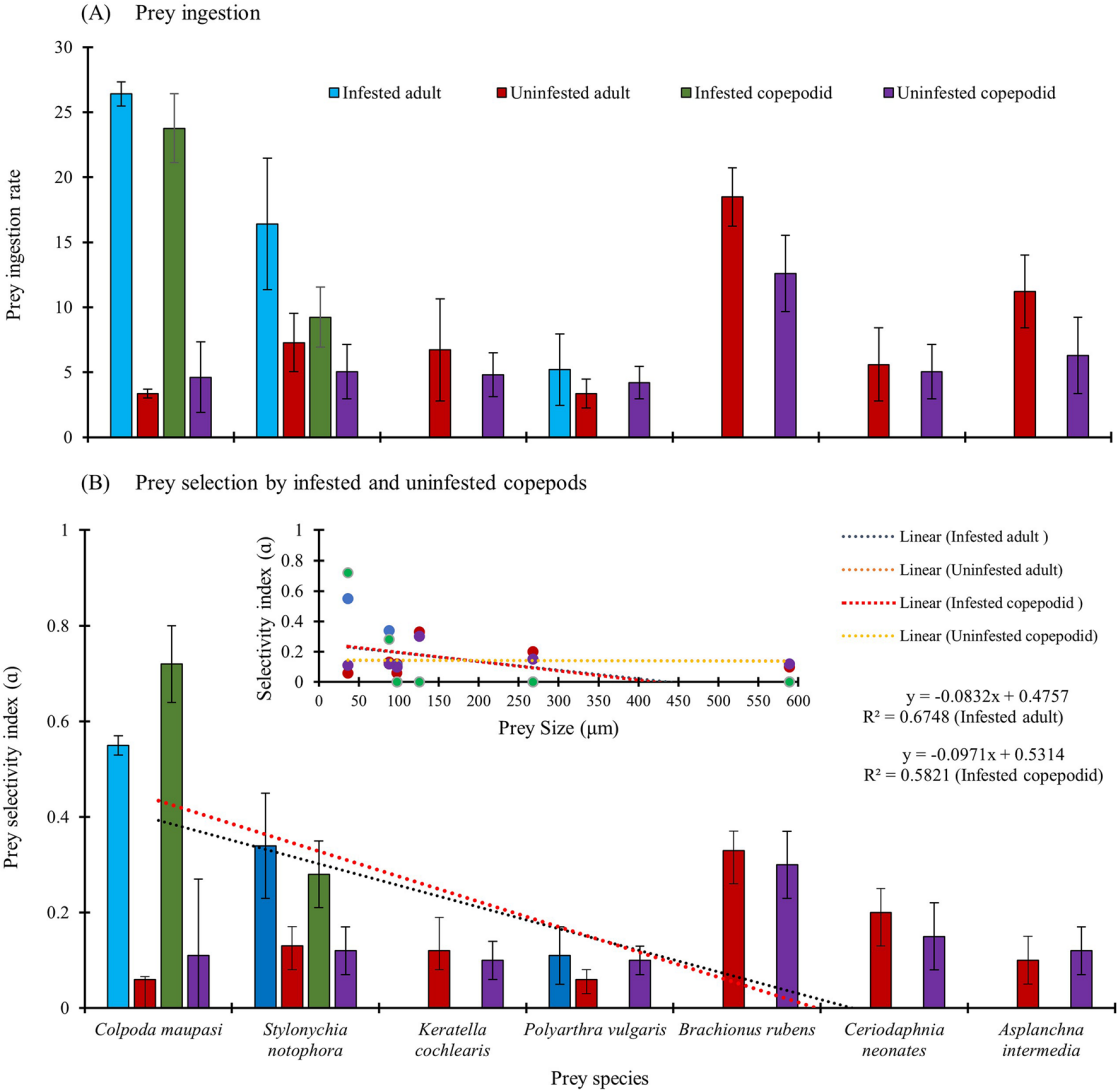
Prey ingestion rate (**A**), prey selectivity index value (ɑ) and prey selectivity as a function of body size (**B**) of *Mesocyclops* given a choice of ciliate, rotifer and cladoceran prey in the laboratory (see Table 1 for prey size).

Seven different types of food species (Table 1) differing in swimming pattern, evasiveness, body size, and taxonomic groups and co-occurring in the same wetland, from where epibionts were recorded. These were used for feeding trials with infested and uninfested adult and copepodid stages of *Mesocyclops*. The length, width and dry weight of prey species, used in the feeding trials are provided in Table 1. All the infested organisms used in feeding trials were colonised by heterotrophic ciliates only. All the prey species were fed to infested and uninfested adults and copepodid stages to assess the impacts of epibiosis on basibiont predation (Fig. 5). The prey preference in copepods differed significantly between infested and uninfested adults and copepodids. The infested adults and copepodids preferred ciliates such as *C. maupasi* (Many’s α = 0.55–0.72 *p* < 0.01 Hotelling’s*T*^2^ test) and *S. notophora* (Many’s α = 0.28–0.34; *p* < 0.01 Hotelling’s*T*^2^ test) over rotifer and cladoceran prey (Fig. 5), whereas, the uninfested individuals of copepodids and adults of *Mesocyclops* preferred the rotifer *B. rubens* (Manly’s α = 0.3–0.33) followed by neonates of the cladocera *C. cornuta* (0.15–0.2; *p* < 0.05 Hotelling’s *T*^2^ test, Fig. 5)*.*

**Table 1:** Length, width and dry weight of different prey species used in the feeding trial experiments values given are mean ± SE (n = 20).

Group	Species	Length (µm)	Width (µm)	Dry weight (µg)	Prey attributes
Ciliophora	*Colpoda maupasi*	36.4 ± 4.01	18.2 ± 2.6	0.005	Sedentary and free-living solitary,
	*Stylonychia notophora*	88 ± 10.85	46 ± 7.3	0.008	Solitary, abundant in eutrophic water
Rotifera	*Keratella cochlearis*	97.8 ± 6.02	60.3 ± 7.4	0.013	Water column, solitary, long spine
	*Brachionus rubens*	126 ± 5.60	64.8 ± 19.8	0.16	Free-living and epizoic on cladocerans. Solitary
	*Polyarthra vulgaris*	97.8 ± 9.58	69.3 ± 21.7	0.90	Solitary evasive movement, long posterior spine
	*Hexarthra mira*				Evasive
	*Filinia longiseta*				Iloricate, with darting movement
	*Asplanchna intermedia*	589.1 ± 26.3	302.8 ± 45.7	1.55	Solitary, free-living, predatory
Cladocera	*Ceriodaphnia cornuta*	268 ± 9.70	152 ± 7.65	1.6	Solitary, free-living
Copepoda	*Mesocyclops* adults				Raptorial movement through power stroke
	Nauplii				Evasive

### Susceptibility of infested and uninfested cyclopoid copepods to fish predation

**Figure 6:**
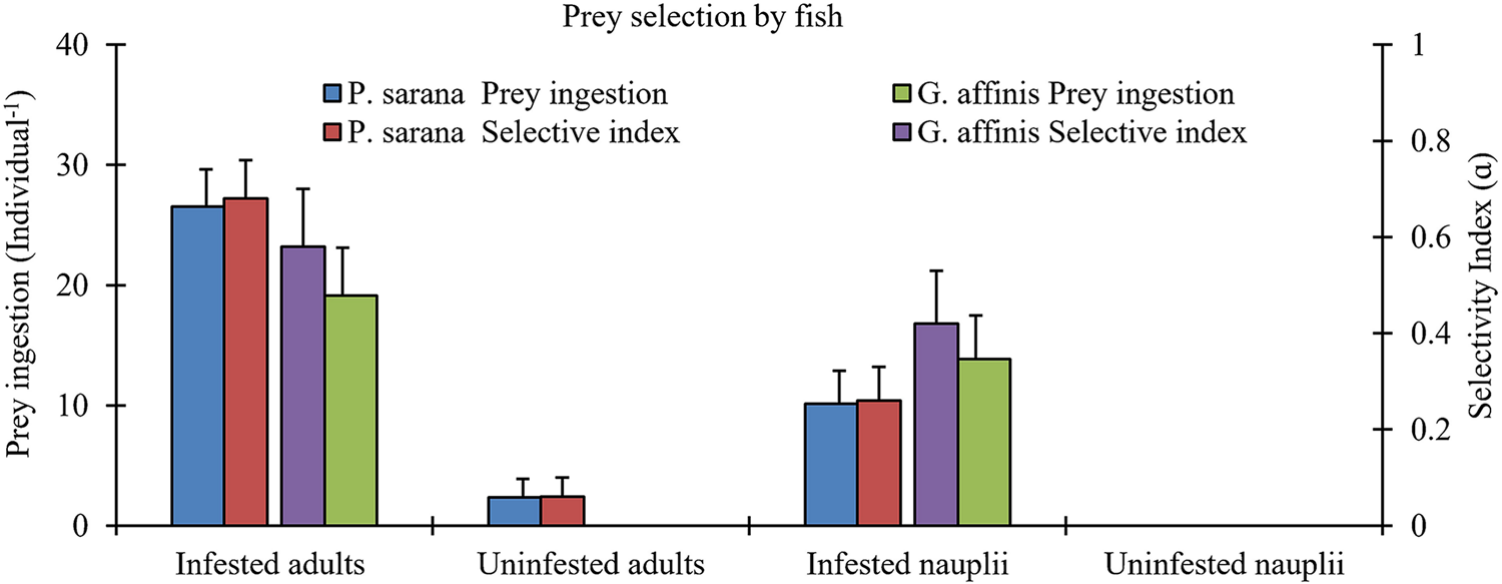
Prey ingestion rate and prey selectivity index value (ɑ) for fish *Punitus sarana* and *Gambusia affinis* given a choice of colonised (burden level for adults: 21.6 ± 3.06; for nauplii 6.3 ± 2.01 epibionts Individual^−1^) and uncolonised adults and nauplii of *Mesocyclops* sp.

The two fish species *P. sarana* and *G. affinis* showed differential prey ingestion rates on infested and uninfested copepods. For fish selectivity experiment the body surface of adult copepods was colonized by 21.6 ± 3.06 epibionts Ind^−1^ and that of nauplii were colonized by 6.3 ± 2.01 epibionts Ind^−1^. Both the fish species showed significantly higher ingestion rates for infested cyclopoid adults and naupliar prey than uninfested individuals (*p* < 0.01, Wilcoxon rank-sum test; Fig. 6). Differences in prey ingestion rates between adult and nauplii of copepods were significant for *P. sarana* (*p* < 0.05, Wilcoxon rank-sum test; Fig. 6) but not for *G. affinis*. *P. sarana* actively selected adult copepods loaded with epibionts (Manly’s α = 0.68 ± 0.08; *p* < 0.001 Hotelling’s *T*^2^ test) and randomly ingested (Manly’s α = 0.26 ± 0.07) nauplii loaded with epibionts, whereas the western mosquito fish *G. affinis* showed avoidance for adult (Manly’s α = 0.06 ± 0.04) and naupliar copepods without epibionts (Fig. 6). *G. affinis* positively selected epibionts carrying adult copepods (Manly’s α = 0.58 ± 0.12, *p* < 0.01 Hotelling’s *T*^2^ test) and nauplii (Manly’s α = 0.42 ± 0.11).

## Discussion

Nutrient rich freshwater ecosystems are frequently providing higher growth and populations of mesozooplankton (rotifers, copepods and cladocerans). However, the plankton communities are often dominated by smaller sized protozooplankton^22,23^. In addition to free swimming protozooplankton, a large number of protozooplankton spends a sedentary life while exploiting resources from different parts of aquatic systems by settling on other free-swimming zooplankton^16^. The external body surfaces of mesozooplankton provides substrate for epizoic protists and rotifers to settle upon^3,10,16,24,25^. The burden of epibionts was associated with area of basionts body surface within a particular group, as the adult copepods were recorded with a higher density of epibionts than the nauplii. This body surface *vs* number of epibiont relationship was not valid for rotifers where larger rotifer *B. rubens, B. plicatilis* had a lower number of epibionts than on the smaller *Keratella*. Colonization on *Filinia, Hexarthra* and *Polyarthra* (Fig. 1) suggest that the evasive behaviour or darting movement did not deter ciliate epibionts from colonization.

Colonisation of ciliate epibionts are observed in various degree of association with different zooplankton taxa and their life stages. According to Bickel et al. slightly alkaline pH values are supporting favourable environments for epibionts^16^ and in our study the average pH was found to be 8.28 ± 0.09. Association of ciliate epibionts on invertebrates are influenced by optimal water quality parameters^4,26,27^. In contrast, we observed no epibiont infestation on *Asplanchna* throughout the study period but the abundance was very low*.* Gilbert & Shröder and Gilbert reported that body surface chemistry of host species facilitates the prevalence of epibionts^28–30^. Cabral et al. reported for the first time ciliate epibiont infestation on different stages of copepods in the Brazilian floodplain^31^ but colonized naupliar stages were never observed.

The present results indicate that swimming behaviour of zooplankton is not a determinant of epibiosis. Instead, the external body surface plays an important role. The predatory rotifer *Asplanchna* was never recorded with epibionts whereas even evasive zooplankton (e.g. *Filinia* and *Polyarthra)*, and those showing darting swimming (cyclopoids) were observed with large numbers of epibionts. *Asplanchna* is an illoricate, translucent rotifer with a saccate body. The body is covered with a thin soft yellowish and proteinaceous cuticle devoid of any spines. The body morphology is changeable in shape as both internal and external organs move. Zooplankton recorded with epibionts have a chitinous cuticle or carapace with spines, and appendages with relatively rough external surfaces. Our field observations support our first hypothesis that the carapace and joint appendages in copepods make this group more susceptible to epibionts under natural conditions. Several epizoans prefer to settle on rough surfaces than on smooth surfaces, and on thoracic appendages of crustaceans rather than on the carapace. Boyan et al. supported our current results emphasizing that basic requirements for attachment of peritrichs to the basibionts are as following: the hydrophilic nature of surfaces^32^, a suitable chemical composition and wettability, protrusion or surface texture/roughness^33^.The epibiont load on *Brachionus* was comparatively less than that of other rotifers but in ovigerous females there was a higher attachment of epibionts. Gilbert explained a major reason for attachment of epibionts on host is dependent on their outer surface chemistry and propensity^29^. The morphological forms and surface secretion of *Brachionus calyciflorus* changes in the presence of *Asplanchna* species as defence mechanism from predation^27^. The congener B. *rubens* does not show any morphological changes in the presence of *Asplanchna,* instead, it gets aggregated or grouped to minimize predation pressure^34^. Congregation and epizoic efficiency of *B. rubens* protect them from copepod predation^35^. Congregation behaviour of *B. rubens* may have given less opportunity to epibionts for attachment. Among rotifers *Polyarthra vulgaris* showed highest infestation with epibionts followed by *Hexarthra*, *Keratella*, and *Filinia,* the most colonised genera with epibionts. Bulut and Saler also reported *Polyarthra* and *Keratella* as most infested taxa by *Epistylis* sp. (Protozoa, Ciliophora) out of 23 zooplankton species studied during spring to winter^35^. Carbal et al. and Kumari et al. reported that ovigerous female rotifers, copepod adults and larval stages such as copepodid and nauplii were more susceptible for colonization by ciliate epibionts due to a large surface area and outer surface chemistry^10,36^.

Epibiosis may offer certain ecolological benefits compared to individuals attached to non-living substrates for instance dispersal, widening their resource base^25^, expansion of habitat-range and protection against competitors and predators^37,38^. On the other hand, intensive epibiosis covers the body surface that may be blocking the basibiont’s body surface receptor cells to perceive signals of info chemicals, in turn affecting escape response to predators, mating response to conspecifics and attack response to prey. Our second hypothesis states that predatory copepods loaded with epibionts would prefer larger prey rather than conspecific individuals without epibionts. The differences in preference between individuals which were uninfested and infested convincingly suggests the impacts of epibiosis on predation by basibionts. However, the preference of uninfested copepods for larger prey (such as rotifers and cladocerans) and that in infested copepods for smaller protistan prey does not support our second hypothesis. Probably, epibiosis restricts the swimming and darting ability of the basibionts as reported from other crustaceans^38,39^. Many copepod nauplii loaded with epibionts did not moult to the copepodid stage even after two weeks of experiment and we recorded 35% to 48% naupliar mortality. Higher epibiont density per unit body surface area of nauplii will have higher ecological repercussions because epibionts restrict the movement of appendages hindering swimming speed and patterns. This would limit the feeding and escape potential of nauplii. Reduction in swimming speed coupled with increased conspicuousness may increase the susceptibility of infested copepods to fish predation. On the other hand, lower mobility and larger body size due to epibionts may protect copepods from size limited, sensory predators like predatory copepods.

We assume that protistan epibionts do not cause mortality directly. Instead, the epibiont loads on the body surface of nauplii make them overstressed and further multiplication of protistan epibionts induce mortality as observed by Wu and Feng^40^.

According to the optimal foraging model the prey profitability (i.e. energy gained/energy spent in ingesting a food item) is an important determinant of prey preference^41–43^. It would be in the best interest of an animal to maximize benefits, so the prey preference in an animal may switch to more beneficial food items^41,44^ based on other ecological conditions such as epibionts in the present case^42^. Body size, conspicuousness and movement patterns are important attributes that determine vulnerability of a prey to predation. Fish larvae are initially mouth gape limited predators and exhibit prey size selectivity, but gradually widen their prey size range as they grow^45^. We suggest that epibiosis-driven increased body size helps basibiont from size selective predators like larvae. However, this increased body size, conspicuousness and decreased movement rate make the epibionts more vulnerable to larger fish.

We observed that the infested copepods were less mobile and unable to raptorially grasp a prey, instead, they switch to more easily capturable smaller prey. Earlier reports suggest that epibiosis provides mimetic protection for the basibiont^4,46,47^. We assume that infested copepods are less preferred prey because protistan infestation and resultant fouling activity would render the copepod less active and distasteful. Therefore, our third hypothesis speculated that visual predators’ such as planktivorous fish would selectively utilize non-infested prey. However, in line with the optimal foraging model the planktivorous ichthyoplankton preferred infested over uninfested copepods. Probably infested copepods become more profitable prey for fish predators as ichthyoplankton obtains more biomass and energy content from epibiotic protists in addition to the copepods. Higher predation pressure on infested prey is attributed to epibionts mediating higher visibility and lower mobility. Therefore, epibiosis does not assist copepods in escaping from predators; instead, it becomes disadvantageous to the basibionts.

## Materials and methods

The present study has been carried out at three levels: (i) field survey to study the relative proportion of epizoic zooplankton in a natural setting; (ii) elucidation of prey selectivity patterns in copepods given a choice of co-occurring infested (by epibionts) and uninfested zooplankton, and (iii) susceptibility to infested and uninfested cyclopoid copepods to fish predation.

### Field sampling

Sampling was carried out in floodplain wetlands of the lower Gangetic basin (25°35′47′′N 85°05′12′′E) at Patna, India. Zooplankton samples were collected on every alternate day from November 2013 to April 2014, using a 35 μm plankton net (30 cm diameter) from the subsurface layer (50–100 cm) water at day-time. A 200-mL subsample of each net tow was preserved with modified Bouin’s fixative at a final concentration of 5%^47,48^. The infested zooplankton species were screened under a dissecting microscope and stained using the Protargol technique^49,50^. Identification of ciliated protist epibionts was done using the identification keys of diverse researchers^51–56^. The relative susceptibilities of zooplankton species were estimated by analyzing epibiont colonies and zooids *vs.* the total zooplankton captured.

### Laboratory experiments

Experimental glass tanks were set up in quadruplicate to conduct experiments in the laboratory. Live zooplankton was collected by filtering 50 L of water from the floodplain wetland of the River Ganga and concentrates were brought to the laboratory, and examined for the presence of epibionts. In the laboratory, 5–15 mL subsamples were examined for zooplankton species composition, ciliate epibiont prevalence (the percentages of zooplankton groups with ciliate epibionts), and epibiont loads (the number of ciliate epibionts per individual zooplankter). Counts from the subsamples were extrapolated to the entire sample, and the average of the four replicates from each site was calculated. These values were used to estimate total epibiont densities (the number of epibionts per cubic meter) in the wetland^6^. Infested (associated with epibionts) and non-infested copepods were separated under a stereozoom microscope (4X). Infested copepods used in the experiments were colonized by colonial stalked ciliate *Epistylis daphniae* Faure-Fremist*.* The dense colonies of stalked ciliates were observed at the base of the urosome, however individual stalked cells were also recorded at the dorsal part of the cephalothorax. Infested and non-infested copepods were transferred to 3-L beakers containing 2-L of filtered wetland water and fed with a mixture of microalgae *Chlorella vulgari*s, rotifers and ciliates.

### Foraging experiment

In nature changes in individual morphology, behavior and/or proximity of other organisms affect their fitness by affecting the foraging patterns of a species or its predators or of both. To elicit the impacts of epibiosis on foraging patterns of copepods and their predators we conducted two separate experiments in the laboratory: (i) epibiosis driven prey preference in copepods and (ii) vulnerability of infested and uninfested copepods to fish predation.

#### Epibiosis drive prey preference in copepods

Prey consumption in infested and uninfested *Mesocyclops aspericornis* was tested in several preliminary experiments. While designing the experiment we sought to answer: (a) what are other co-occurring prey in the natural habitat, where epibiosis on copepods was observed and (b) whether the heavy load of epibionts affects foraging patterns in this cyclopoid. To answer these questions other co-occurring species were collected from the field and acclimatized to laboratory conditions for feeding experiment. Presence of prey species, their size, biomass and important behavioral attributes have been given in Table 1. Both infested and uninfested copepods were sufficiently pre-exposed as all the experimental organisms were simultaneously collected from the same habitat. The prey species were individually counted and introduced in equal proportions to a 400 mL crystallizing bowls, each containing one individual of *Mesocyclops* (either loaded with epibionts or without epibionts). Six individuals of *Mesocylops* were pre-starved for five hours prior to testing. The individuals were used as control (infested) and treatment (uninfested). All the experimental bowls were placed in an unilluminated BOD incubator maintaining a temperature of 25 ± 1.5°C and the copepods were allowed to feed for 3 h duration. Then copepods were removed and prey individuals were counted to estimate the number of consumed rotifers. For each prey type a control without food and without *Mesocyclops* was kept at the same density to get an estimate of multiplication during the feeding duration.

#### Susceptibility of infested and uninfested cyclopoid copepods to fish predation

Two fish species, which are commonly found in the local wetlands, having high values and market demand as ornamentals and vector control, and are also recorded from the habitat where epibiosis on copepods was recorded, the mosquito fish *Gambusia affinis* (Baird and Girard, 1853) and *Puntius sarana* (Hamilton 1822). For this experiment individuals loaded with epibionts and without epibionts of nauplii and adult copepods were used as prey.

Combinations of infested and uninfested individuals of adult copepods and nauplii were provided to either of the fish species in equal proportion. Three hours prior starved fish were used for the experiment and fish were allowed to feed for 60 min duration. All other protocols were the same as in experiment 1. In addition, 10 individual adult cyclopoids and nauplii each carrying epibionts were kept in separate dishes to check if there was any dissociation of epibionts from the copepod during the experimental duration. Fishes were removed from the treatment dishes after 60 min of incubation and remaining prey in each dish were counted.

Infested (associated with epibionts) and uninfested copepods were segregated under a stereozoom microscope (4X). Infested and uninfested copepods were transferred to 3-L beakers containing 2L of filtered wetland water and fed with a mixture of microalgae *Chlorella vulgari*s, rotifers and ciliates.

All experiments, including 5 replicates for each treatment, were conducted in 30 × 20 cm glass troughs containing 2 L autoclaved tap water. A series of short-term experiments was conducted to investigate the feeding rates of the mosquito fish following the protocol used by Kumar et al.^57^. The initial density (Table 2) for each prey type and test duration was chosen based on preliminary feeding experiments such that at experimental termination a substantial number of prey remained unconsumed. The mosquito fish used in the experiments had sufficient exposure to all test prey species prior to testing, since they were cultured using food at different combinations of the same prey species except fish larvae. In total 32 individuals of commercially available fish, including four months old 16 individuals, each of *G. affinis* and *P. sarana* were used for the feeding trials. One individual mosquito fish was transferred from the stock aquaria to the experimental trough 6 h prior to the experiment and deprived of any food. After 6 h of starvation, prey was individually counted and placed gently in each experimental trough. All the experimental protocols were approved by the Research Committee of the Earth Biological and Environmental Sciences School, Central University of South Bihar and the study was reported in accordance to ARRIVE guidelines (https://arriveguidelines.org), guidelines on animal experimentation.

**Table 2:** Epibiont loads (No. of individuals) on ovigerous and non-ovigerous rotifers and adults and larval stages of copepods.

Group of organisms	Counts of Epibiont load/individual			
	Ovigerous		Non-ovigerous	
	Range	Mean ± SE	Range	Mean ± SE
*Keratella cochlearis*	3.0–13.0	5.57 ± 0.60	3.0–19.0	6.37 ± 0.69
*Filinia longiseta*	3.0–5.0	0.32 ± 0.15	4.0–7.0	0.04 ± 0.02
*Brachionus rubens*	2.0–4.0	0.27 + 0.13	2.0–3.0	0.06 + 0.01
*Polyarthra vulgaris*	4.0–34.0	2.07 ± 0.89	2.0–33.0	2.38 ± 0.88
*Hexarthra mira*	4.0–29.0	6.56 ± 0.94	3.0–24.0	3.09 ± 0.85
*Asplanchna intermedia*	0	0	0	0
*Ceriodaphnia*	6.0–23.0	12.53 ± 1.98	7.0–19.0	9.6 ± 2.31
Copepod	2.0–51.0	14.20 ± 3.12	4.0–53.0	14.79 ± 3.47
Copepod larval stages	Copepodite		Nauplii	
	6.0–53.0	18.98 ± 2.60	7.0–29.0	13.74 ± 1.24

#### Statistical analyses

Non parametric Mann–Whitney rank tests were used to determine the differences between expected and observed values of number and dry mass ingested at particular proportions of particular prey types depending upon the two-way ANOVA results^57^.

The difference between expected and actual prey consumption for each prey type choice test in experiment I was analyzed with repeated one-way ANOVA (for details see Kumar et al.^42^). Prey selectivity was calculated using Manly’s selectivity index (α_i_)^41,43,44,58^ modified for a situation in which the predator consumes a substantial portion of the available prey and, hence, prey numbers in the medium decline with time, as in our experiments. The modified formula^54^ is

$$ \alpha = \frac{{\ln \left( {n_{i0} - r_{i} /n_{i0} } \right)}}{{\sum\limits_{j = 1}^{m} {\ln \left( {\left( {n_{i0} - r_{i} } \right)/n_{i0} } \right)} }},\;i = 1,2,3, \ldots m $$

where α_*i*_ is Manly’s α (preference index) for prey type I; *n*_io_ is the number of items of type *i* present at the beginning of a foraging bout; *r*_i_ is the number of items of food type *i* in the consumer’s diet and m is the number of prey types.

Manly’s α_*i*_ values range from 0 to 1. The α_i_ value for non-selective feeding is 0.143 in a multispecies (7 prey types) choice test with copepods and 0.25 in a four prey type choice test (in experiment 2) with fish. Index values above 0.143 in experiment 1 and 0.25 in experiment 2 indicate positive selection (preference) and values below indicate negative selection (avoidance). Deviations in selectivity index values from the value for non-selective feeding were tested for statistical significance using Hotelling’s *T*^2^ test^57^.

## Supplementary Information

Supplementary Information.

## Data Availability

Data can be obtained on request to sumankumari.icar11@gmail.com. It may also be accessed from supplementary material after publication of manuscript.
