# Supplementary material for: Protistan epibionts affect prey selectivity patterns and vulnerability to predation in a cyclopoid copepod
Source: Sci Rep. 2022 Dec 31;12:22631. doi: 10.1038/s41598-022-26004-5 (PMC9805443; doi:10.1038/s41598-022-26004-5)
Supplement: Supplementary file 1 — Supplementary Information. [file 41598_2022_26004_MOESM1_ESM.docx]

**Supplementary Table 1:** Water quality parameters of the wetland, sampled for zooplankton abundance and epibiosis analyses.

**Supplementary Figure 1:**  Zooplankton species infested with epibionts (a) Ovigerous copepod loaded with ciliates (b) adult copepod with ciliates (c) *Moina* (Cladocera; Crustacea) infested with eugelnophytes algae and rotifers (*Brachionus rubens*) as epibionts (d) adult copepod with both the egg sac intact. (e) Copepod nauplii, and (f) Copepodid stage.

**supplementary materials:**

**Supplementary Table1.** Descriptive statistics of water quality parameters of wetland November 2013 to April 2014

| Water quality parameters | Range | Mean± SE |
| --- | --- | --- |
| Water temperature (^0^C) | 16.5-315 | 22.6±2.25 |
| Water depth (m) | 1-1.4 | 1.16±0.05 |
| pH | 8-8.6 | 8.28±0.09 |
| Dissolve Oxygen (mg/l) | 5.7-6.3 | 6.08±0.08 |
| Chloride (mg/l) | 71.2-76.5 | 74.13±0.78 |
| Total alkalinity (mg/l) | 212-272 | 244±10.00 |
| Total hardness (mg/l) | 252-560.5 | 490.5±48 |
| Chlorophyll a (µg/l) | 1.53-2.37 | 1.78±0.13 |
| Chlorophyll b and c (µg/l) | 1.24-1.96 | 1.71±0.10 |

**Supplementary Figure 1:**  Zooplankton species infested with epibionts (a) Ovigerous copepod loaded with ciliates (b) adult copepod with ciliates (c) *Moina* (Cladocera; Crustacea) infested with eugelnophytes algae and rotifers (*Brachionus rubens*) as epibionts (d) adult copepod with both the egg sac intact. (e) Copepod nauplii, (f) Copepodid stage.


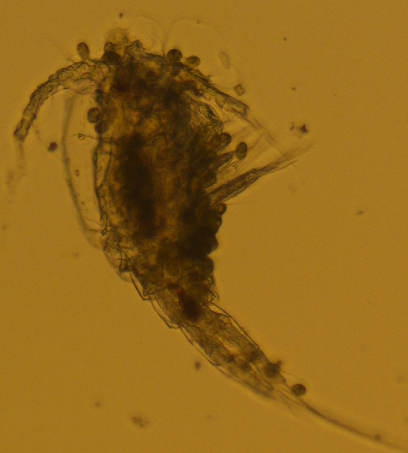

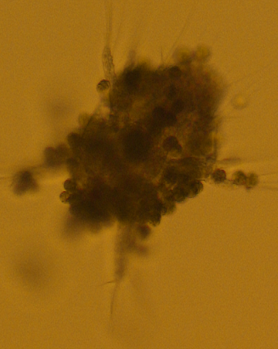

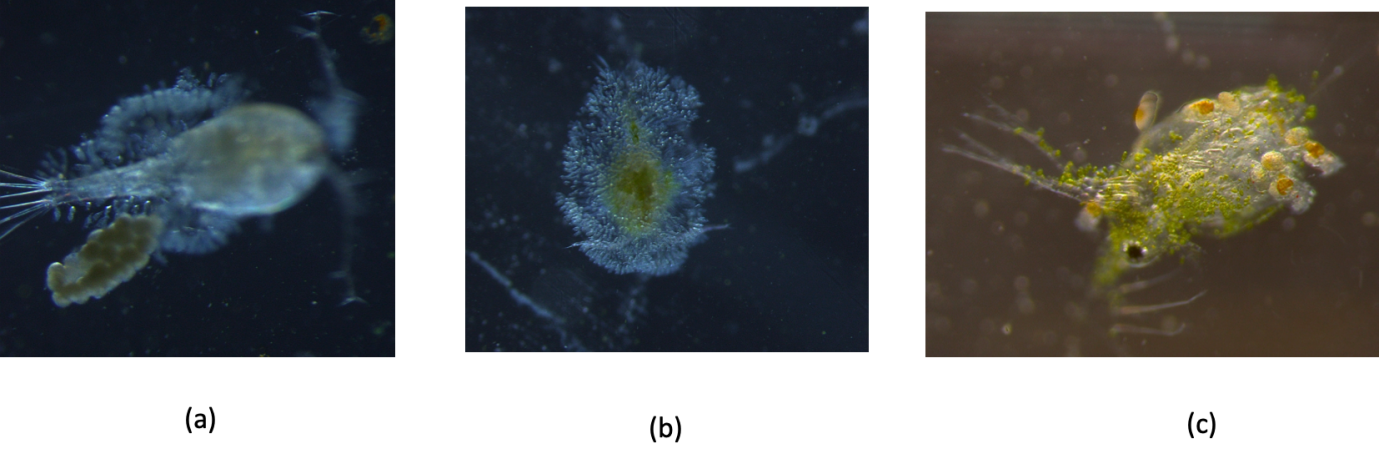


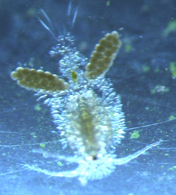


(e))

(f)

(d)


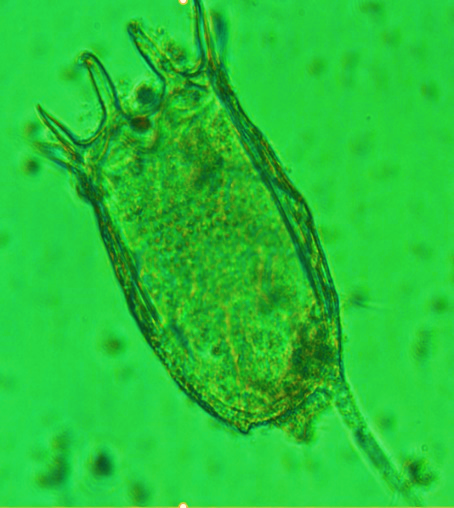


(g)
